# Entrepreneurship Education and Founding Passion: The Moderating Role of Entrepreneurial Family Background

**DOI:** 10.3389/fpsyg.2021.743672

**Published:** 2021-12-01

**Authors:** Younggeun Lee, Andres Felipe Cortes, Minjoo Joo

**Affiliations:** ^1^College of Business and Economics, California State University, Los Angeles, CA, United States; ^2^Welch College of Business and Technology, Sacred Heart University, Fairfield, CT, United States; ^3^Division of Social Sciences, Duke Kunshan University, Jiangsu, China

**Keywords:** entrepreneurship education, entrepreneurial passion, founding passion, entrepreneurial family background, role model

## Abstract

In this paper, we examine the relationship between entrepreneurship education and entrepreneurial passion. Despite the advancement of entrepreneurship education literature and the increasing focus on entrepreneurship education in business schools, we lack empirical exploration on how entrepreneurship education can impact students’ passion for founding new organizations. We hypothesize that students who take entrepreneurship classes would develop high levels of founding passion due to a great perception of skills and abilities that increase positive emotions and decrease negative emotions about the entrepreneurship process. Moreover, we draw on the literature on role models to suggest that students’ entrepreneurial family background (students whose immediate family members are entrepreneurs) strengthens the influence of entrepreneurship education on entrepreneurial passion. Utilizing survey data collected from 160 university students, we found that entrepreneurship education positively influences students’ founding passion and that this relationship is strengthened when students have entrepreneurs in their immediate family.

## Introduction

Countries around the world increasingly acknowledge the importance of promoting entrepreneurship in their populations (Naudé, 2010; Terjesen et al., 2016). The creation of new firms brings about economic growth and creation of employment opportunities. Further, entrepreneurial firms address important societal needs such as solving issues related to public health, poverty, and climate change (Kuratko, 2006; Urbano et al., 2019). As a result, a key emerging challenge of worldwide relevance is promoting entrepreneurial passion, so that more individuals are motivated to establish new firms (Cardon et al., 2009). Part of this important endeavor is being carried out by universities. Many universities are expanding entrepreneurship educational programs at undergraduate and graduate levels by establishing independent Colleges of Entrepreneurship (e.g., Florida State University, United States), Departments of Entrepreneurship (e.g., Hanyang University, Korea), and entrepreneurship centers to promote university-wide entrepreneurial culture (Katz et al., 2014; Morris et al., 2014). Accordingly, research on entrepreneurship education and pedagogy has progressed steadily and explored how entrepreneurship programs can increase students’ entrepreneurial intention (Bae et al., 2014), capabilities (Lee et al., 2018), knowledge (Volery et al., 2013), and inspiration (Nabi et al., 2018).

Despite this important progress, research has not explored how entrepreneurship education can increase students’ passion for starting a business. Considering how entrepreneurship passion drives persistence in new venture efforts (Cardon and Kirk, 2015), employee commitment in new businesses (Breugst et al., 2012), and interest from investors and venture capitalists (Warnick et al., 2018), it is important to understand whether and how entrepreneurship education enhances entrepreneurial passion. This outcome is particularly important as a large portion of university students have chosen to take conservative route of organizational employment, rather than self-employment after graduation (OECD, 2021). Thus, increasing students’ passion toward starting new ventures could lower psychological barriers toward entrepreneurship and nudge motivational factors of students to become nascent entrepreneurs after graduation.

To address this point, we study how entrepreneurship education advances students’ entrepreneurial passion for founding. Entrepreneurial passion refers to intensive positive feelings and central identity toward a specific activity of entrepreneurs such as developing, inventing, and founding (Cardon et al., 2009). Scholars have focused on a specific type of entrepreneurial passion based on the context of study. In this paper, we focus on founding passion to capture students’ passion for starting a new business, which is appropriate for investigating the influence of entrepreneurship education in the context of higher education (Kiani et al., 2020). As such, we do not focus on developing or inventing passion because they are more associated with entrepreneurs or students who are currently managing a firm (Mueller et al., 2017).

We also examine the moderating impact of students’ entrepreneurial family background on the relationship between entrepreneurship education and founding passion. An immediate family member who is an entrepreneur (i.e., entrepreneurial family background) would serve as a role model for students. Family members who are entrepreneurs could give a motivational influence on students by serving as a close example of the entrepreneurship experience (Matthews and Moser, 1996; Wyrwich et al., 2016; Nowiński and Haddoud, 2019). Students not only receive information about the lifestyle of an entrepreneur but also perceive the career success of their role models as potential opportunities and form a positive perception of specific occupations (Scherer et al., 1989).

**Figure 1:**
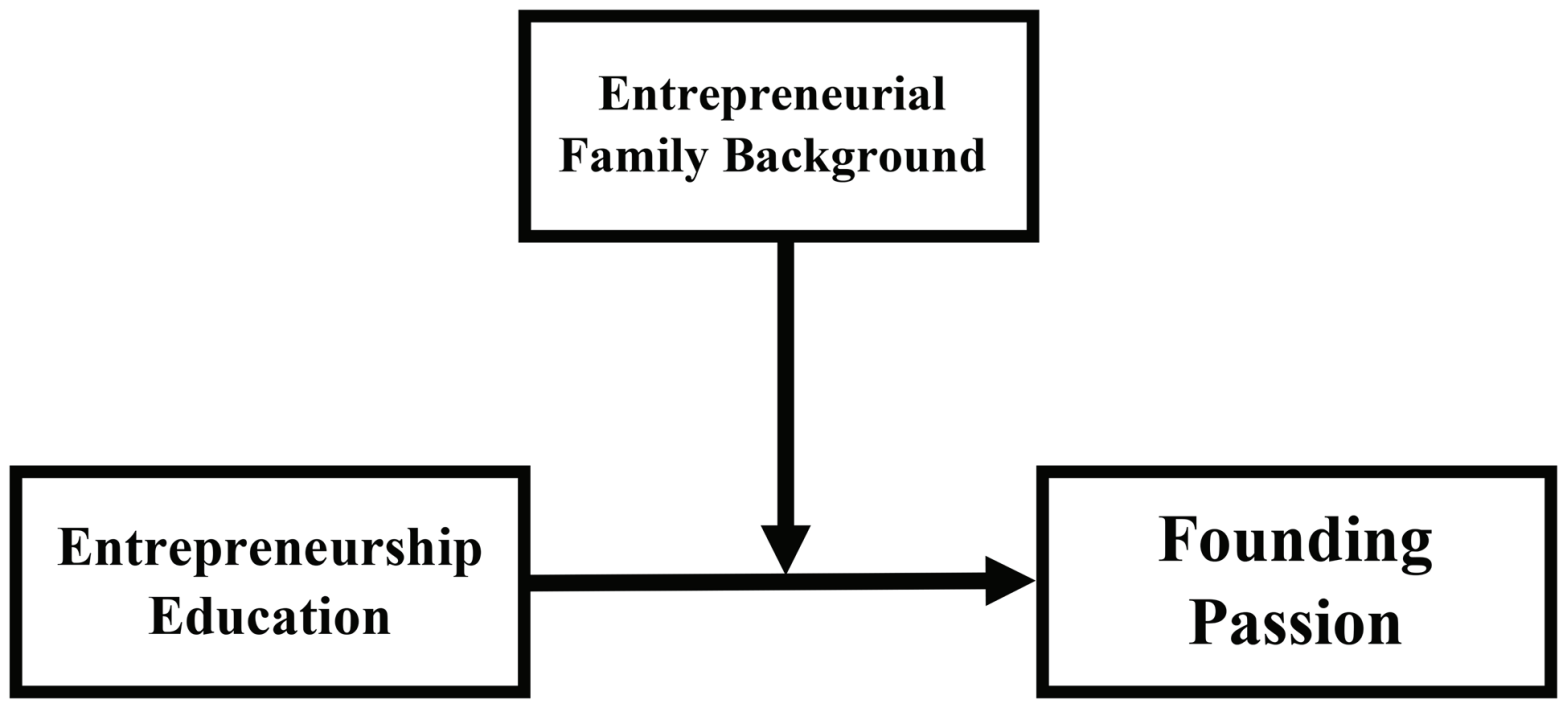
The conceptual model.

The purpose of this study is to examine the influence of entrepreneurship education on the formation of students’ founding passion (Figure 1). As such, we attempt to answer two research questions: (1) how does entrepreneurship education influence the formation of students’ entrepreneurial passion for founding? and (2) how does students’ entrepreneurial family background moderate the relationship between entrepreneurship education and entrepreneurial passion for founding? We test our proposed predictions based on the survey data collected from 160 students at a large Midwestern university in the United States. Our research contributes to the entrepreneurship education literature by studying antecedents of founding passion and highlighting the importance of role models in this process. Specific implications for educators and students are discussed.

## Literature Review

There has been an explosive growth in the number of entrepreneurship degree programs at universities (Morris and Liguori, 2016); as such, entrepreneurship education became the core strategic focus for many universities around the world (Guerrero et al., 2016). Specifically, many schools have implemented entrepreneurship as a major at undergraduate, graduate, MBA, and Ph.D. degree programs and as a minor at various college disciplines (Hornsby et al., 2018; Kuratko and Morris, 2018). Moreover, universities have developed interdisciplinary programs and various pedagogical practices through entrepreneurship centers to promote university-wide entrepreneurial culture (Katz et al., 2014; Morris et al., 2014). Accordingly, understanding the specific outcomes of entrepreneurship education is crucial for educators to distill learning objectives and refine learning materials.

Recent review papers have synthesized the findings on the outcomes of entrepreneurship education and have highlighted its impact on cognitive (e.g., knowledge), skill, affective (e.g., attitude), conative (e.g., intention), and behavioral (e.g., venture creation) factors (Kamovich and Foss, 2017; Nabi et al., 2017; Longva and Foss, 2018). One of the main conclusions of these review papers was that even after considering a wide variety of pedagogical tools, entrepreneurship education programs have generally shown positive consequences for students’ entrepreneurial attitudes and intentions (Nabi et al., 2017).

Despite these important findings and the relevance of entrepreneurship education, scholars have also suggested that it is extremely important to explore unidentified factors in the literature that may strongly influence actual business creation and venturing (Rideout and Gray, 2013). As a result, scholars have encouraged novel research to “go beyond using entrepreneurial intentions as an outcome of the impact” (Kamovich and Foss, 2017, p. 15) and have called specifically for studies on affective outcomes of entrepreneurship education (Longva and Foss, 2018). The premise in these calls is that the intense positive emotions and drive that emanate from entrepreneurial passion can be one of the crucial links between intentions and actual business creation (Nabi et al., 2017), a link that has been contested and is of tremendous importance for entrepreneurship education research (Lorz et al., 2013). Our study therefore addresses these calls by examining the influence of entrepreneurship education on a key affective factor that may drive future business creation: founding passion.

Entrepreneurial passion for founding refers to one’s positive feelings and focal identity toward starting a new firm for commercialization and opportunity exploitation (Cardon et al., 2009). Although only a few empirical studies have employed founding passion (Lee and Herrmann, 2021), scholars empirically have found that founding passion nurtures creativity (Cardon et al., 2013), persistence (Cardon and Kirk, 2015), and entrepreneurial intention (Biraglia and Kadile, 2017), overall suggesting its relevance for entrepreneurship endeavors. The main argument in these studies is that founding passion represents such a central aspect in the new firm establishment process and motivates individuals to dedicate themselves and be persistent toward exploiting opportunities (Cardon et al., 2009). Despite the importance of founding passion to understand nascent entrepreneurs or students who prepare to establish a firm, we still lack theoretical and empirical understanding of how founding passion could be nurtured. Therefore, it is important to determine the antecedent role of entrepreneurship education on students’ passion for establishing a new business.

Along with the increasing need to explore passion in entrepreneurship education research, scholars have also made important calls to consider students’ broader contextual conditions as key players of potential business creation (Liguori et al., 2019; Liguori and Winkler, 2020). One specific variable that has drawn attention is students’ presence of entrepreneurs in their immediate family (i.e., entrepreneurial family background). Early research on entrepreneurial family background mostly focused on the influence of presence of familial role models on entrepreneurial career decisions (Brockhaus and Horwitz, 1986; Scott and Twomey, 1988; Katz, 1992; Krueger, 1993; Matthews and Moser, 1996). Moreover, scholars found that individuals with entrepreneurial family background perceive positive impressions of entrepreneurs (Delmar and Davidsson, 2000) and receive encouragement from family members to become an entrepreneur (Kolvereid, 1996). Over the years, scholars expanded this research stream to examine various effects of entrepreneurial family background such as formation of attitudes toward entrepreneurship (Matthews and Human, 2004), increased social networking opportunities (Anderson and Miller, 2003), emotional and financial support (Aldrich and Ruef, 2006), optimism about gaining necessary skills and resources for starting a firm (Zellweger et al., 2011), and entrepreneurial intentions (Georgescu and Herman, 2020). The core arguments of those studies root from role modeling literature (Matthews et al., 2009), where scholars emphasize learning from immediate family members who are entrepreneurs. It is therefore expected that the effect of entrepreneurship education will be especially pronounced on students’ entrepreneurial passion if students were exposed to entrepreneurship in their families.

Given the importance of entrepreneurial passion for business creation, the lack of empirical exploration about entrepreneurial passion as a key outcome for entrepreneurship education is surprising. Moreover, students’ family background has been identified as a core factor that could create critical variation in student outcomes in entrepreneurship education. Therefore, considering these two factors, we build on the arguments that students’ passion could be developed through efforts (Gielnik et al., 2015) and training (Gielnik et al., 2017) to establish our hypotheses in the next section.

## Hypotheses Development

### Entrepreneurship Education and Founding Passion

We argue that students who take an entrepreneurship class would be able to increase their passion for founding by increasing positive emotions as well as reducing negative emotions associated with establishing a new firm. First, entrepreneurship education focuses on exposing students to key concepts such as opportunity recognition, feasibility analysis, business model, business plan, corporate entrepreneurship, or entrepreneurial financing (Costa et al., 2018; Kuratko and Morris, 2018). By being exposed to these concepts, students learn about the entrepreneurial process from idea generation to venture development and management. Specifically, entrepreneurship classes provide knowledge on factors that early stage entrepreneurs should consider. For instance, students can learn about the concept of business model which in fundamental components (e.g., resources, cost structure, and revenue streams) intertwine to create values (Johnson et al., 2008) that students could benefit from when forming their business ideas.

Through entrepreneurship education, students also become exposed to entrepreneurship case studies, which cover issues such as entrepreneurial financing. Case studies add to the overview of concepts and students are able to learn the practical implications of those concepts through factual case studies. For example, a case study on Kickstarter provides practical knowledge on how early stage entrepreneurs could raise funding from the public. By discussing how a crowdfunding platform works, students compare various fundraising methods for their business ideas such as seed capital, equity funding, and debt financing (Short et al., 2017).

Importantly, entrepreneurship education directly allows students to practice and apply the material with their own business ideas. Specifically, students work on their business plan, pitch their ideas, analyze the feasibility, and peer-review ideas. Through conducting an elevator pitch in the class, students present their new business idea and receive feedback from fellow students on the idea itself (Clingingsmith and Shane, 2018). Then, they establish a feasibility analysis where students learn to access product, market, financial, and organizational feasibility of their ideas. These pedagogical methods lead students to contemplate venture ideas, practice presentations, and evaluate their initial plans. Students who take entrepreneurship classes also learn how to start a firm by completing a business plan. Specifically, by going through key components of a business plan (e.g., industry analysis, development plans, financial projections, and company description), students comprehensively learn the overall picture of a business and study how business ideas turn into an actual firm (Honig and Karlsson, 2004).

Overall, this increased exposure to entrepreneurship concepts and examples and the direct engagement with the planning of their own potential business represents a learning experience that increases students’ confidence in their entrepreneurship skills (Gielnik et al., 2017). The process of starting a new business often seems to be daunting, risky, and full of challenging and uncertain moves. However, the learning experience can help students to develop a set of steps that can be systematized, researched, and planned for a coherent business plan. This can allow students to reduce uncertainty in the entrepreneurship process and have an increased positive perception of their ability to launch a business with greater chances of survival. Accordingly, such experience of progress and accomplishment in a task is associated with the experience of positive emotions, which are important precursors of passion (Vallerand et al., 2003; Cardon et al., 2009). This follows arguments suggesting that individuals can become more passionate in areas and domains in which they believe they can excel (Mageau et al., 2009; Cardon et al., 2017).

Second, considering that starting a new business is a process characterized by obstacles and setbacks, it is possible that students’ positive feelings and emotions about entrepreneurship are counteracted with negative emotions caused by emerging challenges, potentially leading to lower levels of passion. However, research suggests that increased capacity, skills, and knowledge in entrepreneurship can allow students to discard those negative feelings (Bandura, 1991) and potentially understand that emerging obstacles, challenges, and setbacks are an inherent part of the process and should be understood as additional learning opportunities, therefore allowing them to maintain positive emotions associated with passion. Hence, we hypothesize that:

*Hypothesis 1*: Entrepreneurship education is positively associated with students’ entrepreneurial passion for founding.

### The Moderating Role of Entrepreneurial Family Background

Based on the role model literature, we suggest that entrepreneurship education will have a greater influence on entrepreneurial passion for founding for students who have entrepreneurs in their immediate family members. Role modeling refers to the motivational influence of salient individuals on observers through an example and exposure process (Matthews and Moser, 1996; Wyrwich et al., 2016; Nowiński and Haddoud, 2019). Although virtually any individual can serve as a role model for an observer, immediate family members are crucial role models because an observer can directly learn and examine the decisional and behavioral process of entrepreneurs by natural exposure to the immediate family members in their daily lives.

Students perceive the career success of role models as opportunities for them and make a positive perception of those occupations (Scherer et al., 1989). Immediate family members who are entrepreneurs serve as a model that students tend to observe when evaluating their goals and career aspirations (Matthews and Moser, 1996; Nowiński and Haddoud, 2019; Georgescu and Herman, 2020). This increases the possibility that those role models shape students’ attitudes toward founding their own business. Based on their previous exposure to the experience of family entrepreneurs, students will absorb the contents of the class seriously and nurture high levels of passion toward founding. In this regard, we propose that entrepreneurial family background will strengthen the relationship between education and founding passion by allowing students with entrepreneurship role models to reinforce the benefits of entrepreneurship education.

Role models act as a behavioral standard of a student’s social references and provide information on what is commonly done by a group of entrepreneurs (Cialdini et al., 1990). As such, role models would provide an environment where students could enhance their learning obtained from the entrepreneurship class. Specifically, students who have family members as entrepreneurial role models are likely to establish connections between education materials, their role models, and the business started by their role model. This provides an opportunity for the student to develop explanations on why the role model is or was successful with their business and evaluate how their role model dealt or could have dealt with emerging setbacks. These associations can enhance students’ learning by serving as close examples to which they can apply entrepreneurship concepts. As such, we propose that:

*Hypothesis 2*: Students’ entrepreneurial family background positively moderates the influence of entrepreneurship education on students’ entrepreneurial passion for founding.

## Methods

### Sample and Data Collection

This study used survey data collected from 160 undergraduate students at a large Midwestern university in the United States. The survey was distributed in seven different business-related courses, and the response rate was 45.7%. An extra credit was given to the students who filled out the survey. The survey was among several extra credit opportunities that students could choose from. 50% of the respondents majored in business, 49.4% had an entrepreneurial family background, and 33.1% took an entrepreneurship class. The demographics of the survey respondents are summarized in Table 1.

**Table 1:** Demographics of respondents.

Variables	Description	Number of respondents	Percentage
Major department	Business Majors	80	50
	Engineering Majors	29	18.1
	Other Majors	51	31.9
Academic year	Freshman	30	18.8
	Sophomore	12	7.5
	Junior	51	31.9
	Senior	67	41.9
GPA	Between 3.60 and 4.0	7	4.4
	Between 3.20 and 3.59	25	15.6
	Between 2.80 and 3.19	45	28.1
	Between 2.50 and 2.79	45	28.1
	Below 2.50	38	23.8
Entrepreneurial family background	Yes	79	49.4
	No	81	50.6
Entrepreneurship education	Yes	53	33.1
	No	107	66.9

*N=160*

### Measures

*Entrepreneurship education* indicates whether respondents took an entrepreneurship class or not. This measure is in alignment with a recent meta-analysis that showed entrepreneurship education is usually operationalized as a binary variable (Bae et al., 2014). *Entrepreneurial family background* indicates whether students have an immediate family member who is an entrepreneur or not. *Founding Passion* was measured using a four-item scale developed by Cardon et al. (2013); three items captured intensive positive feelings (Cronbach’s alpha=0.86) and one item captured identity centrality for founding activities. To operationalize the construct, the averaged value of three items for intensive positive feelings was multiplied with the value of identity centrality. Based on previous studies (De Clercq et al., 2013; Lee et al., 2018), respondents’ major department, academic standing, GPA, and learning orientation were controlled for. Department was categorized into three majors: business, engineering, and others. Academic standing indicates whether respondents are freshman, sophomore, junior, or senior. Learning orientation was measured utilizing a six-item scale developed by VandeWalle (1997). This scale captured the respondents’ tendency toward new challenges or opportunities for learning (Cronbach’s alpha=0.87). Proactiveness was measured using a two-item scale developed by Lee et al. (2018). This scale accessed respondents’ frequency of preparing for the classes in advance (Cronbach’s alpha=0.57).

### Results

The summary statistics and correlations among variables of the study are displayed in Table 2. Correlations between entrepreneurship education and founding passion was positive and significant (*r*=0.50, *p*<0.01). For Hypothesis 1, a one-way ANOVA was conducted to examine the influence of entrepreneurship education on founding passion; we entered a binary variable for whether or not the student took the entrepreneurship course as a predictor and their founding passion as a dependent variable. As a result, there was a statistically significant difference between two groups [*F*(1, 152)=46.22, *p*<0.001, $\eta_{p}^{2}$=0.23]. Therefore, Hypothesis 1 is supported. Students who took an entrepreneurship class showed statistically significant higher levels of founding passion compared to students who did not take an entrepreneurship class. Tables 3 and 4 display the means and standard deviations of found passion across different conditions.

**Table 2:** Summary statistics and correlations matrix.

Variables	Mean	*SD*	1	2	3	4	5	6	7	8	9
1. Business major^a^	0.50	0.50									
2. Engineering major^a^	0.18	0.39	N/A								
3. Other majors^a^	0.32	0.47	N/A	N/A							
4. Academic year^b^	2.97	1.12	0.20^*^	0.03	−0.23^**^						
5. GPA	3.51	1.14	−0.14	0.12	0.06	−0.05					
6. Proactiveness	3.33	1.29	−0.02	0.18^*^	−0.13	−0.15	0.06				
7. Learning orientation	5.34	0.97	−0.14	0.12	0.06	0.12	0.12	0.35^**^			
8. Entrepreneurial family background^c^	0.49	0.50	0.04	−0.08	0.02	0.07	0.05	0.02	0.07		
9. Entrepreneurship education^d^	0.33	0.47	−0.07	0.05	0.03	0.14	0.06	−0.02	0.18^*^	−0.00	
10. Founding passion	3.24	1.80	0.02	0.00	−0.02	0.12	−0.07	0.03	0.40^**^	0.09	0.50^**^

^*^ p<0.05; ^**^ p<0.01 N=160;

a *Business major, Engineering major, Other majors coded as Yes=1, No=0.*

b *Academic year coded as Freshman=1, Sophomore=2, Junior=3, Senior=4.*

c *Entrepreneurial family background coded as Yes=1, No=0.*

d *Entrepreneurship education coded as Yes=1, No=0*

**Table 3:** Means and standard deviations of founding passion by entrepreneurship education.

		Mean	*SD*
Entrepreneurship education	**Yes**	4.51	1.85
	**No**	2.61	1.4

**Table 4:** Means and standard deviations of founding passion by entrepreneurship education and entrepreneurial family background.

		Entrepreneurial Family Background			
		Yes		No	
		Mean	*SD*	Mean	*SD*
Entrepreneurship education	**Yes**	4.98	1.92	4.06	1.69
	**No**	2.62	1.38	2.59	1.44

**Figure 2:**
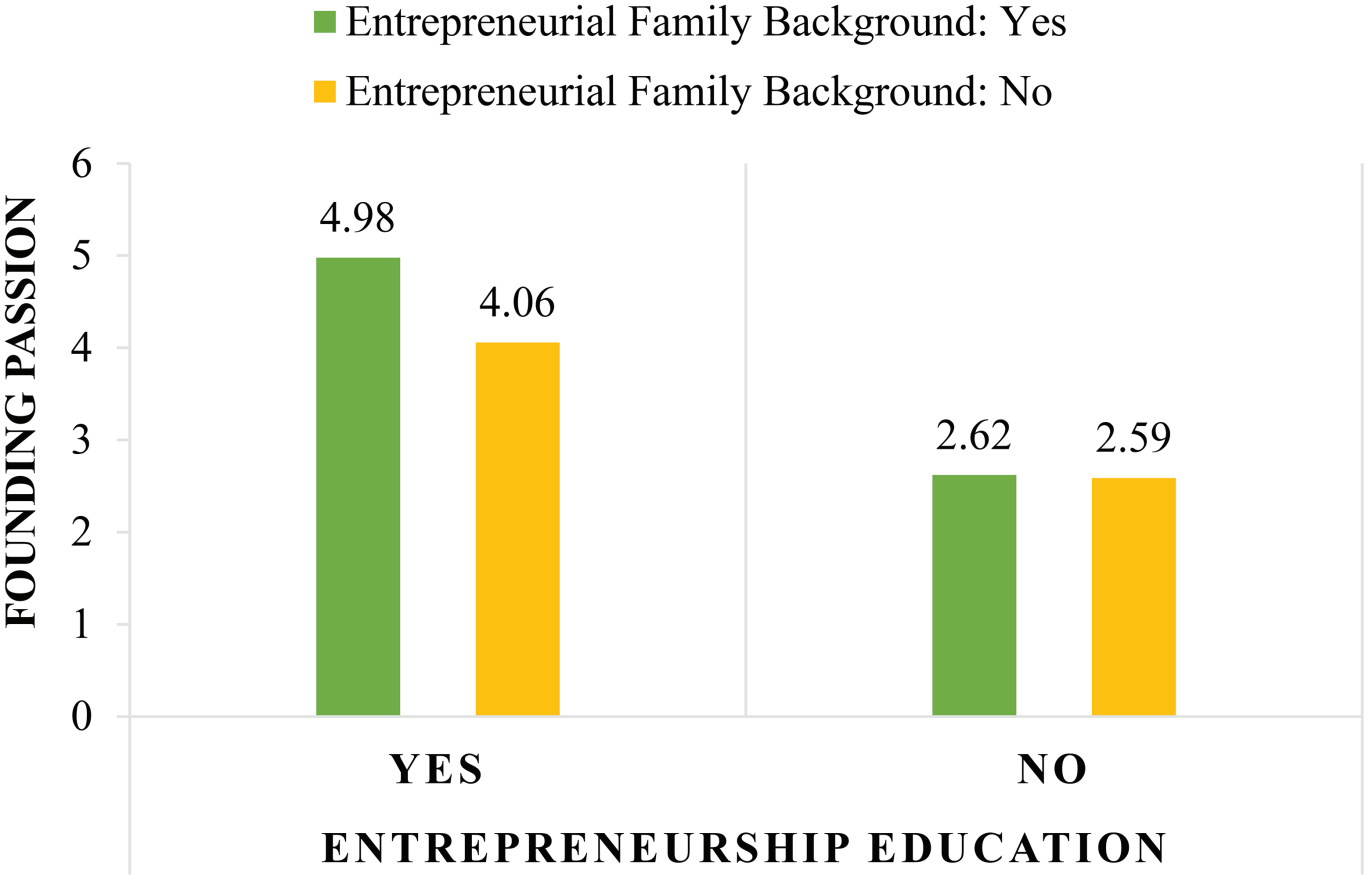
The moderation effect of entrepreneurial family background on the relationship between entrepreneurship education and founding passion.

For Hypothesis 2, a two-way ANOVA was utilized to test the impact of entrepreneurship education and entrepreneurial family background on founding passion. Entrepreneurship education, entrepreneurial family background, and their interaction term were entered as predictors and founding passion as the outcome. There was a statistically significant interaction effect of entrepreneurship education and entrepreneurial family background on founding passion [*F*(1, 150)=4.09, *p*=0.04, $\eta_{p}^{2}$=0.03]. Therefore, Hypothesis 2 is supported. Table 5 presents the results of the two-way ANOVA. The simple effect analysis shows that among the students who took an entrepreneurship class, entrepreneurial family background was significantly related to higher founding passion [*F*(1, 150)=5.40, *p*=0.02, $\eta_{p}^{2}$=0.04]. In contrast, for those who did not take an entrepreneurship class, entrepreneurial family background was not related to one’s founding passion [*F*(1, 150)=0.04, *p*=0.84, $\eta_{p}^{2}$=0.00]. Figure 2 depicts the moderation effect of entrepreneurial family background on the relationship between entrepreneurship education and founding passion.

**Table 5:** Two-way ANOVA results.

	Dependent variable=Founding passion	
	*F*-value	Effect size
**Control variables**		
Business major	2.16	0.01
Engineering major	0.12	0.00
Academic year	0.31	0.00
GPA	3.96^*^	0.03
Proactiveness	2.19	0.01
Learning orientation	28.82^***^	0.16
**Main effects**		
Entrepreneurship education	48.34^***^	0.24
Entrepreneurial family background	3.19	0.02
**Moderation effect**		
Entrepreneurship education × Entrepreneurial family background	4.09^*^	0.03

^*^ p<0.05; ^***^p<0.001; N=160; Effect size=Partial eta squared.

### *Post-hoc* Analyses

We conducted several *post-hoc* analyses to test alternative models. Each dimension of founding passion (i.e., intensive positive feelings and identity centrality) shows different levels of variations over a period of time; intensive positive feelings toward founding tend to variate over time but founding identity centrality tend not to variate after a certain period (Collewaert et al., 2016). Building on this, we attempted to examine whether entrepreneurship education impacts the two dimensions differently by conducting one-way ANOVAs. First, we entered whether or not the student took the entrepreneurship course as a predictor and their intensive positive feelings toward founding as a dependent variable. As a result, there was a statistically significant difference between two groups [*F*(1, 152) =13.95, *p*<0.001, $\eta_{p}^{2}$=0.08]. Students who took an entrepreneurship class showed significantly higher levels of intensive positive feelings toward founding compared to students who did not take an entrepreneurship class.

Second, we used whether or not the student took the entrepreneurship course as a predictor and their founding identity centrality as a dependent variable. As a result, there was a statistically significant difference between two groups [*F*(1, 152)=41.89, *p*<0.001, $\eta_{p}^{2}$=0.22]. Students who took an entrepreneurship class showed significantly higher levels of founding identity centrality compared to students who did not take an entrepreneurship class.

## Discussion

The increasing focus on entrepreneurship programs and initiatives throughout the world is pushing educators to investigate the implications of entrepreneurship education on their students. Thus far, we know that entrepreneurship education has implications for intent and capabilities (Bae et al., 2014; Lee et al., 2018), but we have limited research exploring whether entrepreneurship education can increase students’ founding passion. Exploring this specific outcome is important because founding passion implies one of the core characteristics of entrepreneurs who plan to establish a firm to commercialize and exploit opportunities (Cardon et al., 2009), and only a few studies have examined antecedents of founding passion (Lee and Herrmann, 2021). Furthermore, comprehensive entrepreneurship education should also consider students’ family background and how such background can shape the effectiveness of the material. We take an important step in this direction by exploring how students’ entrepreneurial family background determines their interaction with the content of entrepreneurship courses. Our results supported our predictions: entrepreneurship education increases founding passion, and this relationship is strengthened when students have an immediate family member who is an entrepreneur.

### Theoretical and Practical Implications

Our work has implications for two important areas of entrepreneurship education. First, our work joins other studies that have explored positive outcomes of entrepreneurship education (Bae et al., 2014; Nabi et al., 2018) and have consistently shown that taking entrepreneurship classes can instill the skills and knowledge required for individuals to consider starting a new business (Volery et al., 2013; Lee et al., 2018). However, our study takes a step further by underscoring the critical implications of entrepreneurship education for founding passion, thus bringing an emotional lens to study this phenomenon. This lens is important because passion and positive emotions about the startup process are crucial for individuals who want to start a business, often more so than knowledge or skills (Cardon et al., 2012). This means that although students may feel less uncertainty about starting a new business and perceive they have more knowledge to do so successfully after being exposed to an entrepreneurship class, they do not necessarily feel enthusiastic or eager to do so. We therefore believe that it is very important for an entrepreneurship curriculum to ensure that students’ passion for founding is considered and measured as a key instruction outcome. Moving beyond general entrepreneurship education, it would be important for universities to diffuse university-wide entrepreneurial culture to encourage and foster positive views about entrepreneurship endeavors among various stakeholders such as faculties, staffs, local businesses, and administrators. For instance, entrepreneurship centers could contribute by establishing university-level programs such as pitch competitions, networking fairs, guest speaker series, entrepreneurship camps, and research conferences to help students, faculties, and staffs become familiar with entrepreneurship and lower their negative emotions toward starting a new firm (Finkle et al., 2006; Lee et al., 2018).

Second, our work suggests that some students (those whose immediate family members are entrepreneurs) might develop more founding passion after entrepreneurship courses due to the natural exposure they have had outside of the classroom to the phenomenon of study. This supported the view that entrepreneurial role models allow students to have easily accessible examples to which they can associate class material and reinforce content with a familiar setting, making them to feel more enthusiastic and passionate about the material. This finding suggests that educators should consider different ways of introducing role models to students in the classroom, for example by including in-depth case studies, guest speakers, or biographies. Although role models introduced during a course may not have the same effect as role models who are immediate family members, they can represent the second-best option for some students to take more advantage of the material. This finding also suggests that educators need to take an increasingly complex view of how entrepreneurship material impacts students, mainly by investigating how students’ life experiences and backgrounds shape their interaction with the material.

Our *post-hoc* analyses also provide important implications. Scholars theoretically stated that teaching entrepreneurs to become knowledgeable of the domains that provide them positive feelings is the core aspect to promote their entrepreneurial passion (Cardon et al., 2009; Drnovsek et al., 2016). Moreover, scholars found that two dimensions of founding passion (i.e., intensive positive feelings and identity centrality) displayed different variations after a period of time. Extending on these studies, we conducted *post-hoc* analyses to examine whether entrepreneurship education influences two dimensions of founding passion separately. The results of our *post-hoc* analyses showed similar patterns across two dimensions; entrepreneurship education positively nurtures both intensive positive feelings toward founding and founding identity centrality. Thus, our empirical results indicate that entrepreneurship education has both temporary (i.e., intensive positive feelings toward founding) and lasting (i.e., founding identity centrality) effects on students’ passion. Our findings are in alignment with studies that found individuals’ passion could be developed through efforts (Gielnik et al., 2015) and training (Gielnik et al., 2017).

### Limitations

The limitations of our study can provide important suggestions for future research. First, our work is based on a general entrepreneurship class that was instructed in a specific cultural and economic context. This raises the question of whether founding passion is likely to increase for students who are taking different entrepreneurship classes or for those who are taking similar classes in different universities and countries. For example, with the rising interest in social entrepreneurship (see Cortes and Lee, 2021), it would be interesting to explore whether classes on this topic increase students’ founding passion for businesses with a social purpose, especially in contexts where businesses’ role in alleviating social issues is important. We therefore encourage scholars to conduct similar studies in different contexts to evaluate the validity of our results. Second, our measure for entrepreneurial family background is constrained to the presence of immediate family members who are entrepreneurs, but students may have additional role models who are not in this circle, such as friends, coworkers, supervisors, or more distant relatives; who can impact the way students relate to the material presented in entrepreneurship courses. Future scholars could explore whether these different potential role models have implications for students’ founding passion and compare their influence. In doing so, scholars could also bring new dimensions to this variable by capturing students’ level of exposure to their role model’s entrepreneurship process.

Third, although we employed founding passion as an outcome of entrepreneurship education, students could also develop other types of passion. We especially chose founding passion in the justification that founding passion is the core characteristic of students or entrepreneurs who plan to start a firm. The importance of each type of passion varies in accordance with different roles of entrepreneurs and developmental stages of firms (Cardon et al., 2013). For example, MBA students who are currently managing their own firm or who are in the executive suites might require post-founding passion to expand their firms and advance their own careers. In this regard, we call for future studies on the impact of entrepreneurship education on various types of passion such as developing, inventing (Cardon et al., 2009), harmonious, obsessive (Vallerand et al., 2003), or general work passion (Baum et al., 2001). Lastly, we utilized a cross-sectional survey dataset to test our hypotheses, which limits our understanding of causal effects. We suggest future studies employ experiments or longitudinal datasets to provide further support on the impact of education on passion.

## Conclusion

Our study suggested and found that entrepreneurship education increases founding passion and that this relationship is stronger for students who have an entrepreneurial family background (i.e., students who have an immediate family member who is an entrepreneur). We highlight the importance for entrepreneurship educators to instill positive emotions in the entrepreneurship process and consider how students’ backgrounds can shape learning and founding passion. We hope to encourage more research on this topic.

## Data Availability Statement

The raw data supporting the conclusions of this article will be made available by the authors, without undue reservation.

## Ethics Statement

The studies involving human participants were reviewed and approved by the Institutional Review Board, Iowa State University. The patients/participants provided their written informed consent to participate in this study.

## Author Contributions

YL wrote the first draft of the manuscript and collected the dataset. AFC and MJ wrote sections of the manuscript. YL and MJ performed the empirical analyses. All authors contributed to manuscript revision, read, and approved the submitted version.

## Funding

This work received financial support from Duke Kunshan University.

## Conflict of Interest

The authors declare that the research was conducted in the absence of any commercial or financial relationships that could be construed as a potential conflict of interest.

## Publisher’s Note

All claims expressed in this article are solely those of the authors and do not necessarily represent those of their affiliated organizations, or those of the publisher, the editors and the reviewers. Any product that may be evaluated in this article, or claim that may be made by its manufacturer, is not guaranteed or endorsed by the publisher.
